# Language nonselective lexical access in bilinguals: Input modality matters

**DOI:** 10.1017/S1366728925100928

**Published:** 2026-01-15

**Authors:** Kristi Hendrickson, Anna Sagan, Hector Sanchez Melendez, Jina Kim, Zara Harmon, Stephanie De Anda

**Affiliations:** 1 Communication Sciences and Disorders, University of Iowa, USA; 2 Duke Institute for Brain Sciences, USA; 3 Max Planck Institute for Psycholinguistics, Netherlands; 4 Communication Disorders and Sciences, University of Oregon, USA

**Keywords:** bilingual lexical access, biliterate, eye-tracking, cross-language activation, word recognition

## Abstract

It has been argued that lexical access in bilinguals is language nonselective. However, little is known about how the input modality (spoken or written) affects cross-language activation during listening and reading. The current study characterizes the nature of within- and cross-language competition for spoken and written words in adults who are bilingual and biliterate in Spanish and English. Using a recently developed cross-modality version of the Visual World Paradigm, we found that competition differs for spoken and written words. For spoken words, the auditory stimulus unfolds overtime giving an additional boost to within- and cross-language competition. Conversely, written words can be seen at once, and thus, incremental processing is less of a factor, resulting in less competition within a language and no competition across languages. The findings show that word recognition is fundamentally language nonselective but can behave in selective ways depending on the modality of the input and language experience.

## Highlights

- Study examined within- and cross-language competition in bilingual adults.
- Spoken and written words show distinct competition patterns in the Visual World Paradigm.
- Spoken words showed strong within-language competition in both languages, with written words showing it only in Spanish
- Cross-language competition appeared only for spoken words, not written words.
- Modality and language history shaped how bilinguals co-activate both languages

## 1. Introduction

There is a long-standing debate regarding the extent to which bilinguals access their two languages selectively. The selective access account suggests that bilinguals exclusively activate one language, whereas the nonselective access account posits that both languages are activated in parallel. Decades of research have provided evidence for nonselective access (e.g., De Groot & Nas, 1991; Dijkstra et al., 1999; Dijkstra & Van Heuven, 2002). However, extant research exclusively focuses on spoken or written language using different tasks, which disallows a comparison between modalities. Although similar, spoken and written language each impose differential sensory and cognitive demands. As a result, the degree to which bilinguals access their two languages while listening and reading in one may differ across modalities.

In the current study, we investigate spoken and written language processing using the same experimental method to examine similarities and differences in how bilinguals manage nonselective language access in each modality (listening vs. reading). Theories and methods of word recognition offer a useful framework for uncovering the mechanisms that underlie parallel language activation during listening and reading. Word recognition is a shared fundamental building block for language and literacy and has well-documented models and methods that cut across the spoken and written modalities (Hendrickson et al., 2022; McClelland & Elman, 1986). Here, we investigate the degree to which spoken and written word recognition is language nonselective in individuals who are bilingual and biliterate.

### 1.1. Word recognition in bilinguals

Bilinguals build cross-language associations for spoken words as early as the second year of life. Toddlers are faster at word recognition in the nondominant language when they have a strong vocabulary in the dominant language (DeAnda et al., 2018). Furthermore, there is ample evidence that adult bilinguals activate their languages in parallel while listening and reading in one language (Bijeljac-Babic et al., 1997; Blumenfeld et al., 2016; Brysbaert et al., 1999; Chen & Ho, 1986; Costa et al., 2005; De Groot et al., 2000; De Groot & Nas, 1991; Dijkstra et al., 1999, 2000, 1998; Doctor & Klein, 1992; Grainger, 1993; Grainger & Dijkstra, 1992; Kroll & Stewart, 1994; Li, 1996; Nas, 1983; Preston & Lambert, 1969; Schulpen et al., 2003; Soares & Grosjean, 1984; Van Assche et al., 2009; Van Heuven et al., 1998).

Models of word recognition describe an automatic, incremental and parallel process. Instead of waiting until the end of words to recognize them, proficient listeners and readers build word representations bit-by-bit over time. As a result, a fundamental component of word recognition is competition: given the input (e.g., LIZARD), multiple lexical candidates that are similar to the input (LIVER, WIZARD) are activated and compete for recognition, and those candidates that are not the target are gradually suppressed (Allopenna et al., 1998; Marslen-Wilson, 1987; McClelland & Elman, 1986). Moreover, competition occurs across languages, such that lexical competitors in the nontarget language are activated (e.g., Marian & Spivey, 1999). This dynamic competition among words helps listeners and readers to make rapid and flexible decisions about the identity of the words they hear and read. Together, bilingual research suggests that language activation is largely nonselective, such that both languages are activated to some degree even in cases when only one language is being used.

Though the aforementioned research on bilingual nonselective lexical access was crucial for providing evidence of language co-activation, this research largely utilized priming, lexical decision or neighborhood density tasks, which limit broader interpretations regarding the nature and degree of cross-language activation in bilinguals. This is because such tasks can measure cross-language influence (i.e., does a priming word in one language influence the recognition of a target word in another language), but not whether one language is active while processing the other language (see Hendrickson et al., 2022 for further discussion). Furthermore, these tasks can simulate scenarios akin to code-switching, in which words from different languages are presented in quick succession, likely encouraging cross-language activation (although see studies on the cognate facilitation effect and the homophone effect that have effectively addressed this issue; e.g., Schulpen et al., 2003; van Assche et al., 2009). Indeed, a more stringent examination of nonselective language access examines cross-language activation without language alternation. Finally, these tasks cannot measure how cross-language activation unfolds in real-time.

For spoken words, many of these limitations have been addressed by using eye-tracking in the Visual World Paradigm (VWP), which is a temporally sensitive measure of what words are considered in the milliseconds leading up to word recognition (Cooper, 1974; Tanenhaus et al., 1995). For this task, participants see four images – a target image (e.g., CANDLE), one or more phonologically similar words (e.g., CANDY) and phonologically unrelated items – and are asked to click the picture corresponding to an auditorily presented word, while their eye-movements are tracked. Eye-movements in this paradigm are a proxy for the degree to which a word is activated at any given moment. Typically, participants fixate the target and competitor early because the speech signal is congruent with both. As the word unfolds, participants direct more looks to the target and fewer to the competitor. Participants will look around the screen to locate the target, and unrelated items act as a baseline level of looking. Thus, to show activation, competitor items must be fixated significantly more than unrelated items.

Using the VWP, cross-language activation in spoken word recognition has been demonstrated in bilinguals of many languages: Dutch–English (Weber & Cutler, 2004), Spanish–English (Ju & Luce, 2004; Sarrett et al., 2022), Japanese–English (Cutler et al., 2006), Finnish–French (Veivo et al., 2018) and German–English (Blumenfeld & Marian, 2007). This suggests that cross-language competition is a standard feature of the recognition of spoken words.

### 1.2. Limitations of existing studies

Although a growing number of studies use the VWP in evaluating bilingual word recognition, there are several limitations to the existing line of work on bilingual nonselective lexical access. First, it remains unclear how proficiency and dominance influence cross-language competition. In their seminal study, Marian and Spivey (1999) found that Russian-English bilinguals activated phonological competitors from their L2 into the L1, but not from the L1 into their L2, although, in a follow-up study, they found the opposite effect (Marian & Spivey, 2003). In both studies, cross-language competitors received more looks in both languages; however, the magnitude of the effect differed and did not reach significance. Spivey and colleagues suggest that these between study differences are due to a myriad of factors that are specific to studies of bilinguals: participant selection, language mode and stimulus selection (e.g., ensuring equal phonetic overlap between competitors and target items in both languages). Recently, it was found that when L2 learners (i.e., English speaking university students learning Spanish as a second language as adults) listen in their L2, they robustly activate their L1 (Sarrett et al., 2022). However, activation from L1 to L2 was not tested within this study, leaving further questions about the bidirectional nature of cross-language activation. Although there is some evidence that competition is heightened in cross-language contexts for bilingual adults with higher levels of L2 proficiency (Blumenfeld & Marian, 2013), a bidirectional evaluation of cross-language competition across languages in highly proficient bilinguals is needed to fully describe language nonselective access.

Second, most research tests within- or cross-language competition but not both (Blumenfeld & Marian, 2013; Ju & Luce, 2004; Weber & Cutler, 2004). Addressing both languages in a within-subjects design ensures a more direct comparison of how within- and cross-language competition is managed by bilinguals. Those studies that do test both within- and cross-language competition only test cross-language competition while listening in the L2 (Sarrett et al., 2022). The lack of studies evaluating within- and cross-language competition across both languages creates challenges in reconciling findings across studies with heterogeneous bilingual populations and in building theories of word recognition that reflect the dynamics of bilingual language processing.

Third, prior research focuses on spoken as opposed to written words. Indeed, almost no work in bilingual written word recognition directly investigates what candidates are active and when, and no studies have compared spoken and written word recognition to examine similarities and differences in how bilinguals manage competition in each modality. Recently, the traditional spoken word version of the VWP has been adapted for use with written words and deployed in monolingual populations (Apfelbaum et al., 2023; Gregg et al., 2022; Hendrickson et al., 2022; Kim et al., 2022). In the current study, we use this recently developed written word version of the VWP to directly compare cross-language lexical activation while listening and reading.

### 1.3. The significance of direct comparisons of spoken and written word recognition

In monolinguals, direct comparison of spoken and written word recognition has revealed similarities and differences across the two systems. Specifically, both spoken and written words demonstrate competition and therefore require a characterization of lexical similarity from which to resolve uncertainty and achieve word recognition (Hendrickson et al., 2022). However, for spoken words, the uncertainty is temporal in nature, as the unfolding speech signal gives the beginnings of words an additional boost in activation compared to written words, which are presented as a whole. For written words, spatial rather than temporal uncertainty drives competition, as the reader attempts to identify the target referent.

Although the findings in monolinguals suggest word recognition varies by modality, the lack of studies comparing spoken and written word recognition in bilingual populations is a crucial limitation for several reasons. Research on one modality cannot be applied to the other because spoken and written words develop and are processed differently. For monolinguals, oral language is at least partly acquired by the onset of reading instruction (NICHD Early Child Care Research Network, 2005). For bilinguals in their second language, oral and written language may be acquired simultaneously or sequentially and this may result in differences in language dominance across modalities (Bedore et al., 2012). Dominance within each modality is impacted by quantity and quality of learning experiences across language(s) used at home, school and work (and particularly, the language in which people learn to read). This learning history could lead to differences in the magnitude and timing of cross-language competition for spoken compared to written words.

Importantly, comparing spoken and written words also affords a novel test of nonselective language access from which theoretical models of bilingual word recognition can be refined. First, by employing only spoken words, the degree to which temporary uncertainty of the unfolding signal drives nonselective language access cannot be evaluated. Consider the following example from Sarrett et al. (2022). When participants hear the beginning of the word CHIEF, it is consistent with both CHIEF and CHICLE (gum). Consequently, cross-language activation may be exhibited not because English is activating Spanish, but because the input itself is consistent with Spanish. Thus, due to the nature of the unfolding speech signal, cross-language activation could still be observed without both languages being linked in any way. Examining written word recognition permits a comparison where the temporal unfolding of the input is less of a factor than for spoken words. For written words, competition does not derive from temporary uncertainty, as all letters can be seen at once. Written words provide a comparison to test the role of temporal access to spoken input in explaining language nonselective access.

Furthermore, comparing written and spoken word recognition allows an evaluation of the role of shared forms (e.g., shared orthographies and phonologies) in nonselective language access. The auditory and visual signals carry different amounts of information about which language a given word came from: many languages use highly similar alphabets even when the speech sounds of the spoken language may differ (Shook & Marian, 2013). This may lead to differences in how competition occurs in each modality. Consider a listener hearing an English word (e.g., BOAT): the speech sound /b/ is produced differently in Spanish and English, and this could help listeners activate only the /b/ items in English and suppress those in Spanish (Ju & Luce, 2004). However, this would not help for written words since the letter “b” is identical in English and Spanish and could lead to greater language nonselective access and cross-language competition. Together, comparing spoken and written words evaluates the role of temporary ambiguity and shared representations and forms in language nonselective access.

### 1.4. The current study

The current study will characterize the nature of lexical competition within and across languages for spoken and written words in adults who are bilingual and biliterate in Spanish and English. Cross-language competition from Spanish to English and from English to Spanish is evaluated. We will determine *how* bilingual and biliterate adults recognize spoken words as speech unfolds over time, and written words as reading occurs in the moment. We use a recently developed variant of eye-tracking in the VWP. Participants hear or see a word and click the corresponding picture from a display of four: the target, a phonologically and orthographically similar competitor within- or cross-language, and unrelated items. Eye-movements are measured over time across the four images to reveal the dynamics of lexical competition within and across Spanish and English. The degree to which words from the nontarget language are considered for recognition will test language nonselective access in spoken and written modalities.

#### 1.4.1. Predictions

*Within-language competition*. Consistent with previous work on spoken and written word recognition in monolinguals, we predict that bilinguals will display significant within-language competition in both modalities. However, given that speech unfolds over time, and listeners are thus confronted with moments of temporary ambiguity regarding word identity, we predict there will be more within-language competition in the spoken compared to the written modality. The relative magnitude of within-language competition across languages is less clear. Because participants in the current study have relatively balanced language proficiency in English and Spanish, there may be a similar magnitude of activation for within-language competition in each language.

*Cross-language competition*. A major motivation for lexical competition is the bottom-up uncertainty in the signal: the more uncertainty present in the signal, the more competing words are considered. This could lead to greater cross-language competition for spoken than written words. Conversely, recall that speech sounds may be slightly different across languages (e.g., /b/ in English and Spanish), while for written words, there exists a significant overlap in orthography (e.g., a “b” looks the same in Spanish and English; e.g., Shook & Marian, 2013). As such, for written words, increased uncertainty to language identity due to overlapping forms may lead to more cross-language competition than in spoken word recognition.

In addition to modality, cross-language lexical competition has also been shown to be influenced by language dominance and proficiency. However, research has only focused on spoken words and still the results are mixed. Some studies have shown competition only from L2 to L1, others have found competition only from the L1 to L2, and still others have found that lexical competition interacts with proficiency, such that competition is heightened in cross-language contexts for those with higher levels of second language proficiency (Blumenfeld & Marian, 2013; Marian & Spivey, 1999, 2003; Sarrett et al., 2022). Therefore, whereas we expected to find cross-language competition, it remained unclear whether written or spoken word recognition would demonstrate greater cross-language competition and evidence of language nonselective access, and how dominance and proficiency would be associated with patterns of activation in a group of adult bilinguals with high proficiency but mixed dominance and age of acquisition.

## 2. Method

### 2.1. Participants

Thirty participants (female, n = 21; male, n = 9), with an average age of 21 years were recruited through the University of Iowa’s mass email system, postings on the University of Iowa campus and surrounding areas, and through word-of-mouth. To characterize language experience, proficiency, dominance and use participants completed the Language Experience and Proficiency Questionnaire (LEAP-Q; see Table 1 for results).

**Table 1.** Participant characteristics as collected on the LEAP-Q

Participant characteristics	*n*(%)	*M*(*SD*)
Age in years		22.35(3.17)
Male	9(30)	
Female	21(70)	
English^a^		
English age of acquisition (years)		3.9(3.96)
English speaking proficiency		9.33(.92)
English understanding proficiency		9.6(.89)
English reading proficiency		9.5(.82)
Spanish		
Spanish age of acquisition (years)		.97(2.71)
Spanish speaking proficiency		7.83(1.95)
Spanish understanding proficiency		9.23(.98)
Spanish reading proficiency		7.93(1.72)
Current exposure		
Spanish		21.66(13.54)
English		78(13.75)
Current use		
Spanish		44.5(24.47)
English		51.83(25.58)
Current reading		
Spanish		25.13(25.72)
English		74.8(25.68)
Cultural identification^b^		
American	21(70)	
Latino/a/x	7(23)	
Hispanic	7(23)	
Mexican	4(13)	
Ecuadorian	2(7)	
Guatemalan	1(3)	
Colombian	1(3)	
Italian	1(3)	
Mexican American	1(3)	
Nicaraguan	1(3)	
Puerto Rican	1(3)	
Venezuelan	1(3)	

a One participant reported French as a 3^rd^ language acquired in adulthood with minimal current exposure and use.

b Participants could list more than one cultural identity.

The LEAP-Q is a widely used self-report measure of language history and proficiency in bilingual populations (Kaushanskaya et al., 2020). The questionnaire includes the ages of acquisition of each language, length of immersion in different contexts and estimates of proficiency in speaking, reading and understanding among other variables. The LEAP-Q has excellent reliability and validity (Marian et al., 2007).

Results from the LEAP-Q showed that participants acquired Spanish earlier than English (*t*(31) = 2.5, *p* = .02; see Table 1). However, their self-reported proficiency in speaking (*t*(31) = 3.7, *p* = .0008) and reading (*t*(31) = 4.5, *p* = .0001) was better in English than Spanish. We found no difference in self-reported levels of understanding across language (*t*(31) = 1.4, *p* = .2). Current exposure and reading also favored English, although self-ratings of current speaking suggested relatively balanced use of English and Spanish. Together the LEAP-Q results suggest that participants as a group demonstrated some dominance in English in their reading but spoke both languages with similar frequency at the time of test and showed high proficiency in both languages (i.e., high self-ratings across speaking, understanding and reading in both languages as shown in Table 1). Notably, although reading and exposure to English was rated high at the time of testing, participants as a group had a significantly earlier age of acquisition of Spanish.

### 2.2. Visual world paradigm

#### 2.2.1. Experimental design

**Figure 1.**
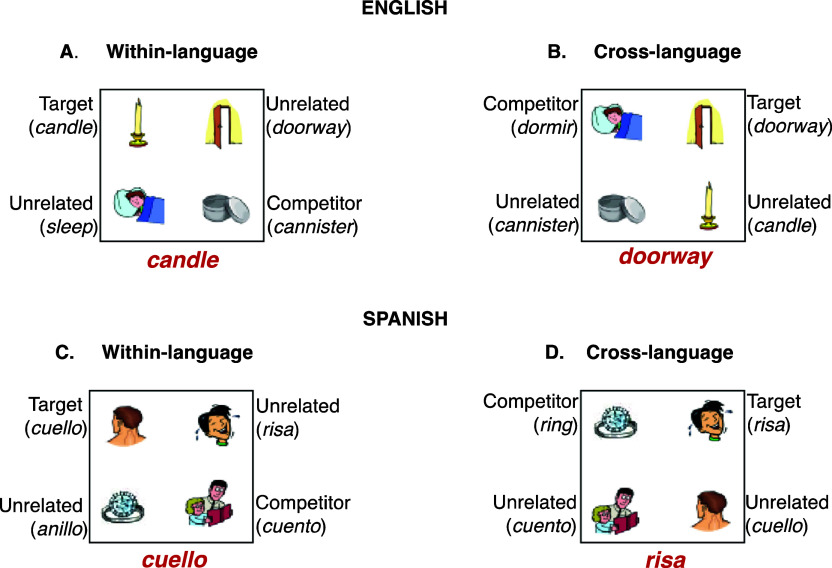
Example trials across four experimental conditions. *Note*: English within-language (A), English cross-language (B), Spanish within-language (C) and Spanish cross-language (D).

There were four conditions: two testing within-language competition (English to English, e.g., CANDLE-CANNISTER; Spanish to Spanish, e.g., CUELLO [neck]-CUENTO [story]) and two testing cross-language competition (English to Spanish, e.g., DOORWAY-DORMIR [sleep]*;* Spanish to English, e.g., RISA [laugh]-RING) (see Figure 1). For each condition, there were 20 word pairs with phonemic and orthographic overlap word-initially. To ensure proper phonological and orthographic overlap, we used the following selection criteria: (1) no words with diacritics, (2) no cognates (e.g., telephone-télefono) and (3) only words that contain letters that were present in both languages. It must be noted that we endeavored to select words containing phonemes found in both languages; however, achieving perfect balance proved challenging. Even when phonemes and vowels appeared similar, differences in contrasts, subtle distinctions and phonotactic coarticulation between languages made complete balance unfeasible. All words were highly familiar, and across the four conditions, there was no significant difference in word frequency per million (*F*(3, 76) = .02, *p* = 0.996), and percent phonological (*F*(3, 76) = 0.41, *p* = 0.75) and orthographic overlap (*F*(3, 76) = 0.32, *p* = 0.81) between word pairs.

To ensure that any cross-language competition effects were not a result of frequent language switching, English and Spanish trials were blocked and counterbalanced across participants such that within each block, the target was presented in only one language. Word pairs were combined into sets of four, such that one word pair acted as unrelated items for another word pair. Word pairs from the cross-language English to Spanish condition were combined with word pairs from the within-language English to English condition in the English block (e.g., DOORWAY*-*DORMIR [sleep]-CANDLE-CANNISER), while word pairs in the cross-language Spanish to English condition were combined with word pairs from the within-language Spanish to Spanish condition (e.g., RISA [laugh]*-*RING*-*CUELLO [neck]*-*CUENTO [story]) in the Spanish block (see Supplementary Material). Because word pairs acted as unrelated items for other word pairs within a set, it was important to combine word pairs that were phonologically, orthographically, and semantically unrelated. Thus, we ensured that word pairs within a set did not, (1) overlap in the first three letters or phonemes, (2) contain any vowels that overlapped in the same slot (e.g., *token –* toad), (3) contain translations that were similar to the other word in the pair (e.g., VELA [candle] – VEIL[velo]) and (4) contain semantic or featural overlap.

During the experiment, each word in a set appeared as the target an equal number of times. This resulted in two different trial types: competitor present and competitor absent. For example, for the word set DOORWAY*-*DORMIR [sleep]-CANDLE-CANNISTER in the English block, when DOORWAY, CANDLE or CANNISTER was displayed as the target, the trial was a competitor present or Target-Competitor-Unrelated-Unrelated trial (see Figure 1). However, when DORMIR appeared as the target, the translation SLEEP was presented to preserve the language block, thus making all other words unrelated and creating a Target-Unrelated-Unrelated-Unrelated (TUUU) trial. These sets of four words always appeared together.

**Figure 2.**
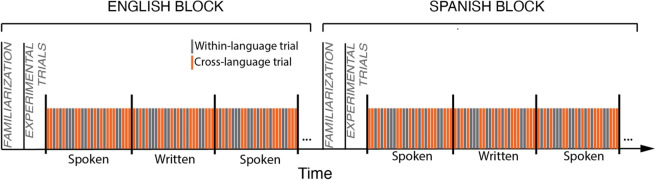
Experimental design diagram. *Note*: The experiment was blocked by language and counterbalanced across subjects. Within each language block, there was a familiarization phase in which participants were presented with each image one at a time and read the word aloud. Within each language block, trials were further blocked by modality (20 modality blocks [24 trials each], 10 spoken and 10 written). Modality blocks were counterbalanced across subjects. Between each modality block there was a drift correct and short break. Within modality block, within- and cross-language trials were randomized.

Within each language block, modality was blocked (and counterbalanced across participants) such that every 24 trials there was a brief drift correct, and then the presentation modality switched from spoken to written (see Figure 2). For each language block, 10 item sets were repeated twice per condition resulting in 320 trials (10 item sets × 4 items/set × 4 language conditions × 2 repetitions), and 40 trials per condition.

#### 2.2.2. Stimuli

*Visual stimuli* were images matched for style and salience developed using a standard lab protocol (e.g., Apfelbaum et al., 2011, see Figure 1 for examples). Each image underwent a rigorous selection process, in which multiple candidate pictures for each word were sourced from a commercial clip art database. Only words that were deemed highly picturable were chosen. Subsequently, a committee of five English–Spanish bilingual lab members selected the picture deemed most suitable to represent each word across languages. When necessary, images were edited to eliminate extraneous features and ensure the use of prototypical colors and orientations. Moreover, efforts were made to equate images in terms of style and visual prominence, with final selections validated by a senior lab member well-versed in the VWP. The majority of images utilized in this study were drawn from a database established through this rigorous protocol and have been employed across multiple research studies (McMurray et al., 2010). *Spoken words* were recorded by an early simultaneous adult bilingual female speaker with native proficiency in Mexican Spanish and Standardized American English with exposure to both language varieties since birth. Five Spanish–English bilingual lab members noted no discernible additional accent in either language. Participants received no information regarding the speaker’s bilingualism or background. All words were amplitude normalized to 70 dBA. *Written words* were presented in black Times New Roman font, size 40. The stimuli used in this study are available on Open Science Framework (https://osf.io/dx86s/).

#### 2.2.3. Procedure and recording

At the beginning of the experiment, participants were seated in front of the computer monitor and the eye-tracker was calibrated and validated using a 9-point procedure. Before each language block, there was a familiarization phase, in which participants saw each word and the corresponding picture one at a time (see Figure 2 for experimental design diagram). Finally, two practice trials were administered to ensure participants understood the task.

On each trial, four pictures appeared on the computer monitor. Pictures were 300 × 300 pixels and appeared 50 pixels vertically and horizontally from the edges of a 17 in. computer monitor running at 1280 × 1024 pixels. Picture location was counterbalanced across trials, such that all word-types and conditions (target, competitors [English-within, English-cross, Spanish-within, Spanish-cross] and unrelated items) appeared equally in each location. A blue dot appeared in the middle of the screen and 500 ms later turned red. Participants were instructed to click the red dot at which point the dot disappeared, and the target word was presented. Participants then clicked the corresponding picture. For spoken words, the word was played through over-the-ear noise cancelling headphones at a comfortable volume. For written words, the orthographic form was presented in the center of the screen for 100 ms followed by a backward mask (#######) for 100 ms to limit visual aftereffects (for additional reasoning regarding presentation time, refer to Hendrickson et al., 2022).

#### 2.2.4. Analytical approach

We conducted six analyses for data collected using the VWP. While our primary focus was on discerning the dynamics of competition as a function of language and modality, analyzing target responses and fixations offers valuable insights into how this competition translates into meaningful differences in word recognition. By analyzing target responses and fixations, we probed whether the introduction of a competitor from a distinct modality and language impacted the speed and accuracy of target word recognition. Thus, we first report findings that examined the accuracy and reaction time (RT) of the mouse-click responses and the speed of target fixations by Language (English, Spanish) and Condition (within- or cross-language).

We then directly examined lexical competition using two complementary and well-documented metrics from the looking data – overall proportion looking and peak fixations – and compared these metrics between competitors and unrelated items. First, we report findings of overall proportion looking which is calculated by averaging proportion fixations after the word was heard or read agnostic to time. Second, we report proportion fixations at their maximum (i.e., peak height). Although both measures gauge the magnitude of competition, they are dissociable and complementary. Consider a fixation curve characterized by low kurtosis, indicating a distribution with less pronounced tails and flatter, wider curves compared to those with higher kurtosis, which have more peaked shapes. Such curves may yield high overall proportion looking but relatively low peak values. Therefore, utilizing both measures allows us to capture fixation curves that exhibit gradual buildup over time as well as those with rapid fixation development and swift inhibition. If effects are evident in the overall proportion looking analysis but not in the peak height analysis, it may be inferred that these effects emerge later in processing. Overall proportion looking and peak height variables were examined with the within-subjects factors of Word-Type (competitor, unrelated), Condition (within, cross) and Language (English, Spanish).

## 3. Results

### 3.1. Target mouse-click responses

Both response accuracy and RT (log-transformed) were subjected to two repeated measures ANOVAs, one for each modality (spoken and written) with the within-subjects’ factors of Condition (cross, within) and Language (English, Spanish).

#### 3.1.1. Target accuracy

**Figure 3.**
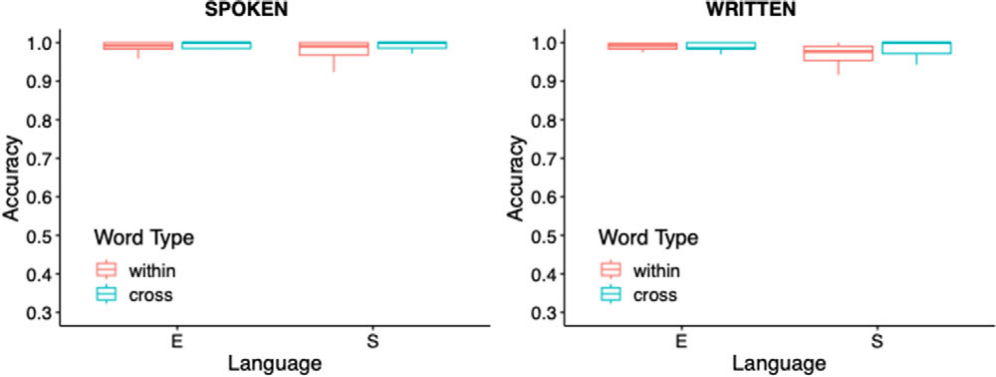
Accuracy to click the target image by Modality (spoken, written), Language (English [E], Spanish [S]) and Condition (within cross). Each box represents the interquartile range (IQR) of the data, with the horizontal line inside the box denoting the median. *Note*: The y-axis does not start at 0 to emphasize the variation within a narrow range of values.

For *spoken* words, there were no main effects of Condition (*F*(1, 29) = 1.48, *p* = 0.234) or Language (*F*(1, 29) = 1.54, *p* = .22), nor was there a significant interaction (*F*(1, 29) = .421, *p* = .52) (see Figure 3).

For *written* words, there was no main effect of Condition (*F*(1, 29) = .068, *p* = .79), and no Condition by Language (*F*(1, 29) = .28, *p* = .60), although there was a marginal main effect of Language (*F*(1, 29) = 3.75, *p* = .063), such that participants were more accurate at recognizing English compared to Spanish words.

#### 3.1.2. Target reaction time

For *spoken* words, there was a main effect of Condition (*F*(1, 29) = 26.62, *p* = .0001, η^2^G = .01), such that participants were significantly faster at responding on cross-language (M = 1317.48, SD = 366.92) compared to within-language trials (M = 1367.20, SD = 349.09) (*p* < .0001, *d =* .14). However, there was no main effect of Language (*F*(1, 29) = 1.33, *p* = 0.25), nor was there a significant interaction (*F*(1, 29) = .58, *p* = 0.45).

For *written* words, there was a main effect of Condition (*F*(1, 29) = 4.46, *p* = 0.04, η^2^G = .004). Similar to spoken words, participants were significantly faster at responding when reading on cross-language (M = 1163.53, SD = 257.41) compared to within-language trials (M = 1203.30, SD = 359.29) (*p* = .025, *d =* .13). Contrary to the results with spoken words, there was also a main effect of Language (*F*(1, 29) = 7.28, *p* = .012, η^2^G = .049). Participants were faster at reading English (M = 1295.89, SD = 194.16) compared to Spanish words (M = 1388.79, SD = 434.00) (*p* = .0005, *d =* .42). There was no significant interaction (*F*(1, 29) = 1.03, *p* = 0.32).

### 3.2. Eye fixations

For analyses of eye fixations, we calculated the proportion of looks to targets, competitors and unrelated items every 4 ms from the onset of the spoken or written word. Our goal was to examine the pattern of lexical activation leading up to successful word recognition. Thus, only those trials in which the participant selected the correct image were included in the analyses of the fixation data. It is important to note that differences in the input modality of the signal between spoken and written words may differentially affect participants’ ability to fixate the images early in the trial. To illustrate, for written words (and not spoken words) the input modality of the stimulus and the measurement are the same (visual) and this may change the sequence of fixations. This makes a direct statistical comparison of competition across modalities challenging (Hendrickson et al., 2022). Thus, our a priori statistical plan did not call for an explicit statistical test comparing modalities, and we focus here on the profile of competition within a modality and leave comparisons for interpretation of results in the discussion.

#### 3.2.1. Target fixations

**Figure 4.**
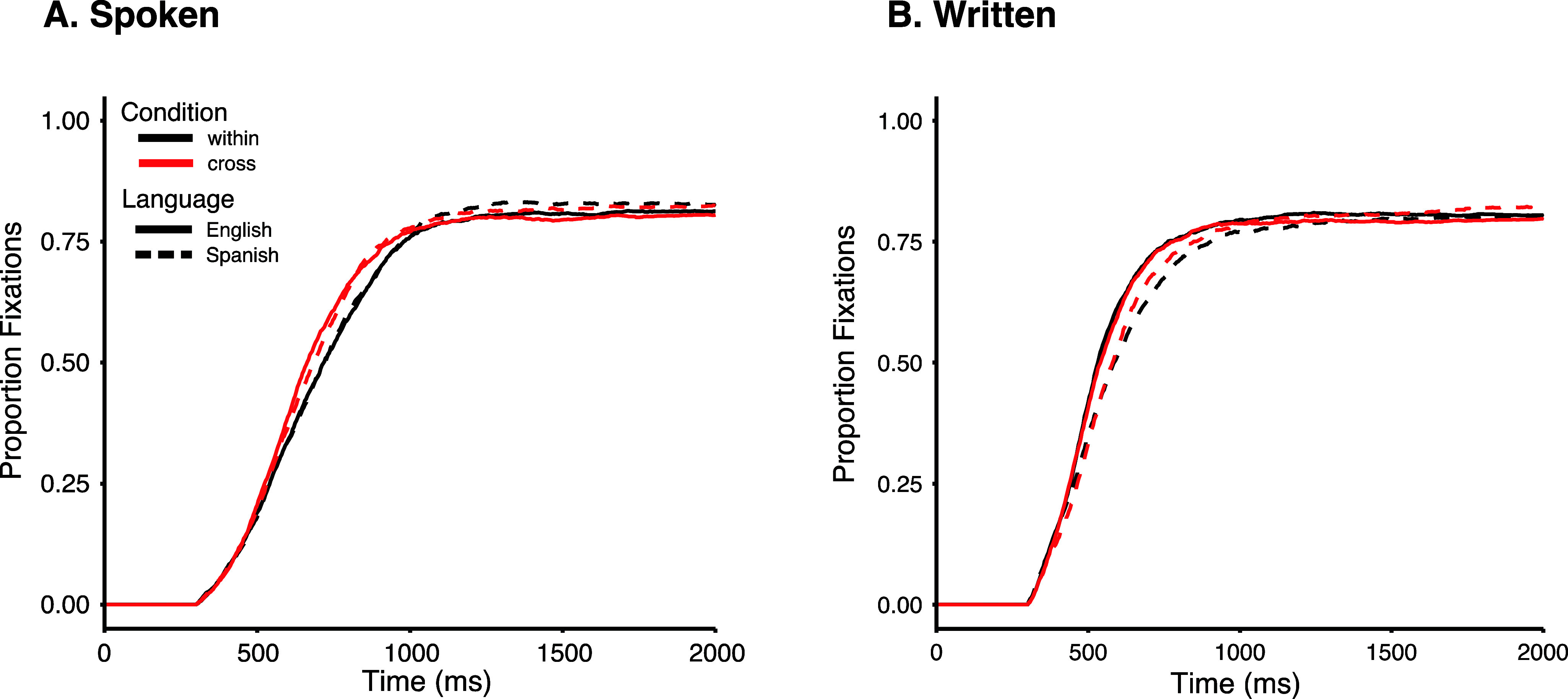
Target fixations over time. Proportion fixations to Targets by Condition (within, cross), Language (Spanish, English), from spoken (A) or written (B) word onset.

Although our primary goal was to examine the dynamics of lexical competition, we also examined the timing of target fixations to obtain another speed of processing measure to complement the accuracy and RT findings (see Figure 4 for fixation curves). Target looks during the VWP are a proxy for the speed with which individuals recognize spoken and written words. To obtain measures that quantify the timing of target fixations we fit logistic functions that model fixations to the target for each participant’s data using nonlinear curve fitting (Seedorff et al., 2018). Fixations were fit with a four-parameter logistic function with time on the x-axis, and separate parameters for the upper and lower asymptotes, the crossover (the time at which fixations were halfway between asymptotes) and the slope (the derivative at the crossover). Fits were conducted separately for each participant, language and condition (236 curves had excellent fits [R^2^ ≥ 0.95], and 4 curves had good fits [.9 < R^2^ ≤ .95]). From these logistic functions, we derived two measures of timing: the slope reflects the speed at which fixation builds whereas the cross-over point reflects any overall delay in the time course of fixations (a shift in time; McMurray et al., 2019).

*Target fixation slope*. For *spoken words*, there was a significant main effect of Condition (*F*(1, 29) = 20.41, *p* = .0001, η^2^G = .068), in which participants were faster to fixate on targets on cross-language compared to within-language trials (*p* < .0001, *d* = 0.53). There was no main effect of Language (*F*(1, 29) = .413, *p* = .53) nor a significant interaction (*F*(1, 29) = 2.48, *p* = .13). For *written words*, there were significant main effects of Condition (*F*(1, 29) = 5.12, *p* = .031, η^2^G = .009), similar to spoken words, targets were fixated faster on cross-language compared to within-language trials (*p* < .0001, *d* = 0.18). There was also a main effect of Language (*F*(1, 29) = 25.51, *p* = .0001, η^2^G = .01), such that English words were fixated faster than Spanish words (*p* < .0001, *d* = 0.65). However, the interaction was not significant (*F*(1, 29) = 3.99, *p* = .055).

*Target fixation crossover*. The results for crossover were similar to the slope analysis. For *spoken words*, there was a significant main effect of Condition (*F*(1, 29) = 26.71, *p* = .0001, η^2^G = .051), where target fixations occurred earlier for cross-language compared to within-language trials (*p* < .0001, *d* = 0.46). There was no main effect of Language (*F*(1, 29) = .937, *p* = .34), nor was there a significant interaction (*F*(1, 29) = 1.51, *p* = .22). For *written words*, there was a main effect of Language (*F*(1, 29) = 23.14, *p* < .0001, η^2^G = .11), in which English words were fixated earlier than Spanish words (*p* < .0001, *d =* 0.71), but unlike for slope, there was no main effect of Condition (*F*(1, 29) = .099, *p* = .76) and no interaction (*F*(1, 29) = .001, *p* = .97).

In sum, the speed of target fixations was associated with different factors for spoken compared to written words. For spoken words, target fixations only differed by condition. Participants processed the target word faster when confronted with a cross-language competitor compared to a within-language competitor. This begins to suggest that there was greater competition for within-language words, which resulted in a delay in activating the target word compared to those that appeared with cross-language competitors. Written words also exhibited faster target word fixations on cross-language compared to within-language trials, but unlike spoken words, this effect was only observed for slope and not crossover, suggesting that the difference in timing between cross- and within-language trials is attenuated in the written versus spoken modality. In addition, for written words but not spoken words target fixations also differed by language. Participants were significantly faster at recognizing English compared to Spanish words when reading.

#### 3.2.2. Competitor fixations

**Figure 5.**
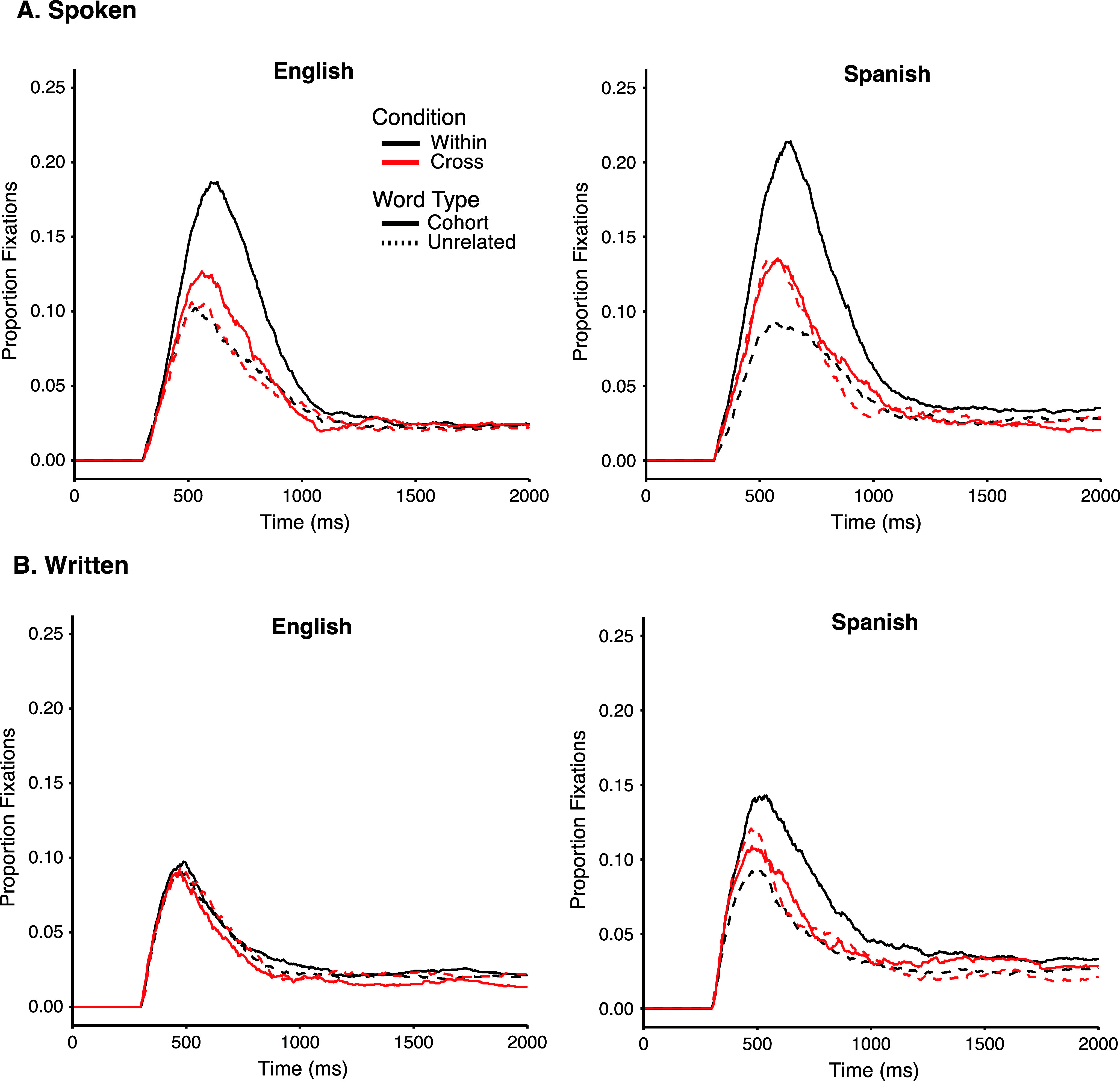
Fixations to competitors and unrelated items over time. Proportion fixations to competitors and unrelated items by Language (Spanish, English) and Condition (within, cross) from spoken (A) or written (B) word onset.

Whereas target fixations describe activation of the target word, competitor fixations reveal activation of potential lexical candidates during recognition. To determine whether there was significant competition as a function of Language and Condition, we conducted two sets of analyses (overall proportion looking and peak fixations). In all analyses, we operationalized competition as a significant difference in looks to the competitor compared to the unrelated baseline (see Figure 5 for competitor and unrelated fixations). Trials in which there were no competitors on the screen (i.e., TUUU trials) were removed from competitor analyses. This was because on these trials, there may still be indirect competition via the translation equivalent that is not commensurate with the more direct cross-language competition we are seeking to examine. For example, on the TUUU trial in which SLEEP is the target presented with DOORWAY, CANDLE and CANISTER, it is plausible that activation of the translation of SLEEP (*dormir*) may induce competition with DOORWAY. Thus, TUUU trials were dropped to ensure more interpretable results across competitor analyses.

*Overall proportion looking to competitors*. To first examine overall effects across time, we calculated the average proportion looking (log transformed) from 250 to 1250 msec post-word onset. We analyzed proportion looking in two repeated measures ANOVAs, one for each modality (spoken, written) with the within-subjects’ factors of Word-Type (competitor, unrelated), Condition (cross, within), Language (English, Spanish). For significant two-way and three-way interactions that include Word-Type, we conducted post hoc paired *t*-test with a Bonferroni adjustment leading to statistical significance at the *p* < 0.025 level.

For *spoken* words, there were significant main effects of Word-Type (*F*(1, 29) = 150.23, *p* < 0.0001, η^2^G = 1.58) and Condition (*F*(1, 29) = 39.83, *p* < .0001, η^2^G = .062), and a significant Word-Type × Condition interaction (*F*(1, 29) = 105.27, *p* < 0.0001, η^2^G = .102). There was no significant main effect of Language (*F*(1, 29) = 3.35, *p* = .078), and no Language × Condition (*F*(1, 29) = 2.12, *p* = .16) or Word-Type × Language × Condition interaction (*F*(1, 29) = 2.61, *p* = 0.12).

To examine the significant Word-Type × Condition interaction, pairwise comparisons compared Word-Type (competitors and unrelated items) for each Condition (within, cross) collapsed across Language. Looks to competitors were significantly greater than unrelated items within language (*p* < .0001, *d* = 1.67), and across language (*p* = .011, *d =* .25).

*Peak fixation to competitors*. We used peak height derived from the fixation data as another measure of the magnitude of competition. Peak height corresponds to the proportion of fixations at their maximum level and has high test–retest reliability (*r* = .70; Farris-Trimble & McMurray, 2013). To derive peak height, fitted Gaussian-like curves were applied to the raw fixation data (McMurray et al., 2010). To evaluate goodness of fits, we measured R^2^ values and visually examined the observed data compared to the estimated curves for each subject. There were 480 fits in all (30 participants × 2 Word-Types [cohort, unrelated] × 2 Conditions [cross, within] × 2 Languages [English, Spanish]). In the fitting stage, 219 curves had excellent fits (*R*^2^ ≥ 0.95), 250 curves had good fits (0.8 ≤ *R*^2^ ≤ 0.95) and 11 curves had fits that were fair (*R*^2^ < 0.8; although no fit was <0.75).

**Figure 6.**
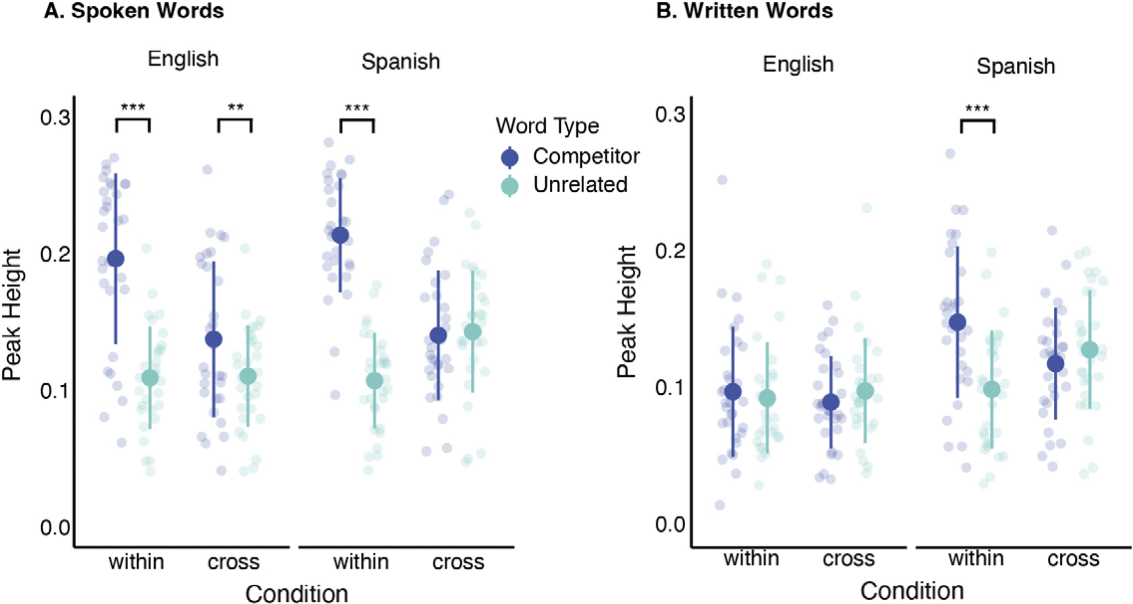
Peak height for spoken (A) and written words (B) by Language (English, Spanish), Condition (cross, within) and Word Type (competitor, unrelated). ***p < .0001, **p < .01.

We analyzed peak height for spoken and written words separately in two repeated measures ANOVAs with the within-subjects’ factors of Word-Type (competitor, unrelated); Condition (within, cross) and Language (English, Spanish). For *spoken* words, there were significant main effects of Word-Type (*F*(1, 29) = 131.35, *p* < .0001, η^2^G = 0.26), Condition (*F*(1, 29) = 37.47, *p* < .0001, η^2^G = 0.064) and Language (*F*(1, 29) = 3.53, *p* = .019, η^2^G = 0.028), and significant Word Type × Condition (*F*(1, 29) = 112.26, *p* < .0001, η^2^G = 0.18) and Word Type × Condition × Language interactions (*F*(1, 29) = 9.085, *p* = .0005, η^2^G = 0.018). We ran a series of post hoc pairwise comparisons (with a Bonferonni-adjusted alpha value of .025) to examine the interaction of Word Type, Language and Condition. There was a significant difference between competitor and unrelated items for English competitors within- (*p* < .0001, *d =* 1.69) and cross-language (*p* = .006, *d =* 0.56). However, for Spanish, only within-language competitors showed significant effects (*p* < .0001, *d =* 2.76; see Figure 6).

For *written* words, we found significant main effects of Word-Type (*F*(1, 29) = 6.32, *p* = .018, η^2^G = 0.011) and Language (*F*(1, 29) = 18.66, *p* < .001, η^2^G = 0.13), and significant two-way Word Type × Condition (*F*(1, 29) = 14.40, *p* = .0007, η^2^G = 0.043) and Word Type × Language (*F*(1, 29) = 9.03, *p* = .005, η^2^G = 0.015) interactions, as well as a significant three-way Word Type × Condition × Language interaction (*F*(1, 29) = 7.38, *p* = .011, η^2^G = 0.018). Post hoc pairwise comparisons revealed that there was a significant difference between competitor and unrelated items only for within-language Spanish competitors (*p* < .0001, *d =* 0.99).

## 4. Discussion

In the current study, we used a modified version of the VWP to address how bilingual and biliterate adults negotiate two language systems and recognize spoken and written words. We first discuss how language and condition influenced the speed and accuracy of target word recognition by modality. Next, we discuss similarities and differences in within-language competition by language and modality. Finally, we discuss results on cross-language competition by language to inform theories of nonselective lexical access that consider the nature of the input signal (listening vs. reading).

### 4.1. Speed and accuracy of target word recognition

Based on self-report (LEAP-Q), participants in the current study acquired Spanish earlier than English, were relatively balanced in their current use of each language, but were English-dominant when reading. Indeed, there was no significant difference in self-reported proficiency in understanding of English and Spanish, although participants reported that their reading skills were significantly better in English.

These differences in dominance across modality were also reflected in the speed of target word recognition during the VWP. That is, a moderate English-dominance effect was observed during reading but not listening to words. Specifically, both RTs to click the target and target fixations were mediated by language and modality, such that participants were faster at recognizing English compared to Spanish words only when reading (with medium-effect sizes; .4 ≤ *d* ≤ .71) This underscores the notion that dominance within each modality can differ based on experiences with language and literacy (Bedore et al., 2012). Whereas participants learned both Spanish and English early in life (on average before 4 years of age), their literacy experiences differed, a fact which revealed dominance effects across self-report (LEAP-Q) and word recognition (VWP).

### 4.2. Within-language competition

#### 4.2.1. Spoken words

Consistent with our predictions, bilinguals displayed significant within-language competition in English and Spanish during spoken word recognition. This is in part a byproduct of the unfolding speech signal which results in increased uncertainty about word identity at any given moment and an increased reliance on the beginning of words (Allopenna et al., 1998; Hendrickson et al., 2022). Given that all lexical competitors in the current study overlapped at word onset, competition was robust for Spanish and English words in the spoken modality.

#### 4.2.2. Written words

For written words, within-language competition was only evident when reading in Spanish. Although numerically written English competitors were fixated more than unrelated words, this did not reach significance. Ostensibly, these results are contrary to results with English monolinguals that show significant activation of written competitors (Hendrickson et al., 2022). However, prior work used only monosyllabic words, while the current study employed multisyllabic words. Recent research suggests that multisyllabic competitors that begin the same display weaker activation than monosyllabic competitors that begin the same because there is less proportion overlap between competitors (Hendrickson et al., 2020, 2022; Magnuson & Simmons, 2020; Simmons & Magnuson, 2018), which may explain our failure to find significant within-language competition for written English words in the current study.

Why then did Spanish demonstrate significant within-language competitor activation for written words, and English did not? We find three interpretations most likely. These findings may be due to differences in orthographic transparency between languages. While the English orthographic system is opaque, Spanish orthography is transparent (i.e., close letter-sound correspondence). Recent work by Kim et al. (2022) shows that phonology is robustly activated even during familiar word reading. Thus, the close correspondence between phonology and orthography in Spanish may work in tandem leading to greater within-language competition when reading in Spanish compared to English in the present study.

The within-language competition effect in reading Spanish words may also reflect language dominance and proficiency. Participants reported stronger reading proficiency in English than in Spanish. Thus, it is possible that Spanish showed within-language competition because there is less proficiency in this language compared to English. In general, highly proficient readers may resolve competition quickly, an effect that was shown in English as the dominant language for literacy.

Finally, as previously mentioned, recent work on *spoken* word recognition in monolinguals suggests that bisyllabic words display more competition than monosyllabic words (Hendrickson et al., 2020, 2022; Magnuson & Simmons, 2020; Simmons & Magnuson, 2018). We sought to control for syllabication, however, Spanish words are naturally longer than English words and this was reflected in our stimuli. A one-way (by-item) ANOVA comparing the number of syllables for words by Condition (English to English, English to Spanish, Spanish to English, Spanish to Spanish), revealed a significant main effect of Condition (*F*(3,116) = 15.683, *p* < .0001, η^2^G = .289). Post hoc *t*-tests (corrected for multiple comparisons using a Bonferroni-adjusted alpha of .008 [.05/6 comparisons]) showed a significant difference in the number of syllables between the English to English and Spanish to Spanish conditions (*p* < .0001) and the English to Spanish and Spanish to Spanish conditions (*p* < .0001). No other comparisons were significant.

Thus, it is possible that the greater number of syllables in the Spanish to Spanish condition compared to the English to English condition resulted in the observed within-language competition differences for written words found in the current study. However, we consider this interpretation less likely. The aforementioned finding of greater competition in bisyllabic compared to monosyllabic words has only been found for spoken words in English monolinguals. The condition that most closely resembles this extant work in the current study is the within-language condition in the spoken modality, in which we found no significant difference between languages.

### 4.3. Cross-language competition

#### 4.3.1. Spoken words

We found evidence for cross-language competition when listening in English and Spanish. Yet, based on a comparison of the two competitor analyses (overall proportion looking and peak height), the relative timing of these effects differed. The overall proportion looking analysis showed competition across languages, with greater activation of competitors in within-language versus cross-language trials. Indeed, there was no three-way interaction that included language, suggesting that the cross-language effects from English to Spanish and from Spanish to English were similar. However, the overall proportion looking analysis is agnostic to the timing of effects because it collapses fixations across time. Conversely, the peak height analysis takes time into consideration because it is a measure of the proportion looking at the time in which fixations reach their peak. Indeed, for the peak height analysis, there was a significant three-way Word-Type, Condition, Language interaction, such that cross-language effects were only present when listening in English. Considering both analyses, it was found that when listening in English, Spanish competitors are activated early (at and around the peak), while when listening in Spanish, English competitors are activated after the peak.

What accounts for this difference in the timing of cross-language effects between languages is not immediately clear. It cannot be attributed to differences in syllables, as there was no significant difference in the number of syllables in the two cross-language conditions. Another possibility related to unrelated looks: looks to unrelated items were higher than expected for spoken Spanish words in cross-language trials, which may have masked earlier cross-language effects. Examination of individual stimulus sets revealed that six sets exhibited elevated looks to unrelated items. However, on closer inspection of these sets we did not uncover any clear artifacts or anomalies that could account for the pattern. A further possibility involves characteristics of the bilingual speaker herself. Although she was an early and highly proficient Spanish–English bilingual speaker with no discernable accent (see Section 2.2.2), it is possible that she had some subperceptual differences in pronunciation across languages due to differences in language learning history. For example, although the speaker maintained high proficiency in both languages, English was the dominant language used in daily life compared to Spanish at the time the stimuli were recorded. These nuances in bilingual language learning history, proficiency and use may lead to variations in phonetic production, resulting in phonetic overlap between words in different languages. This overlap can increase competition during word recognition, as similar-sounding words from both languages activate competing lexical representations. However, considering that multiple bilinguals perceived the speaker to have a typical accent in each language and study participants were not provided with any information about the speaker’s background or identity, including whether it was one or two speakers, it is improbable that the characteristics of the speaker influenced the current results.

If this effect is not attributable to any of the aforementioned factors, it may reflect a genuine property of bilingual lexical processing related to the language learning history of the participants: Spanish was learned significantly earlier than English on average in this group of participants (Spanish learned at birth vs. English learned at age 4 years), giving Spanish L1 status despite the relatively balanced proficiency and use among both languages and the English dominance in reading and exposure at the time of data collection. Although the bilingual participants acquired both languages beginning in childhood and reported use of Spanish and English equally as adults, English was nevertheless learned sequentially and 4 years later than Spanish on average. The early years of oral language development, during which word-recognition processing begins, would have been characterized as Spanish-dominant – monolingual Spanish to be more precise – as the participants reported acquiring Spanish at birth and English at age 4 on average. Bilingual toddlers also show unidirectional cross-language interactions in early oral language development, such that they are faster at recognizing words in their nondominant language when they have a large vocabulary in the dominant language (DeAnda et al., 2018).

This is also instantiated in models of language acquisition that suggest that the first language (L1) bestows linguistic structure for learning in the second language (L2; Mayberry, 2007). That the participants quickly consider Spanish competitor words when hearing English ones ultimately does not affect recognition: target recognition was equal across languages. However, the precision of the VWP reveals that word recognition in adults may reflect latent aspects of early language learning history and experience, such that Spanish words continue to support English word recognition. Indeed, word recognition in adulthood is sensitive to early language learning conditions. It has been shown that age of acquisition modulates the amplitude of the P300 component in spoken word recognition (Tainturier et al., 2005).

Previous research on cross-language competition for spoken words is mixed. Some studies have shown competition only from L2 to L1 (Spivey and Marian, 1999; Sarrett et al., 2022), while others have found competition only from the L1 to L2 (Marian & Spivey, 2003). Given the fact that all participants in the current study had high levels of proficiency in both languages, the current results are perhaps most in line with previous evidence that competition is heightened in cross-language contexts for bilingual adults with higher levels of second language proficiency (Blumenfeld & Marian, 2013).

Taken together with the extant research, the current results suggest that whether cross-language activation occurs depends on several factors: language mode, stimulus selection, participant selection, language history and modality. Language mode (how much the context primes a particular language) likely affects the degree to which languages are co-activated (Marian & Spivey, 2003). Although participants in the current study were tested in both languages in a single experiment session, language was blocked and participants took a break between blocks. Marian and Spivey (2003) also blocked language to reduce language co-activation and found cross-language effects only from L1 into L2.

Another explanation for the mixed results across studies is due to stimuli selection and experimental design. For instance, some studies include word pairs with minimal phonological overlap (NUN – NARANJA [orange]), which may water-down language co-activation (Blumenfeld & Marian, 2013). A component that was unique to the current study was controlling for phonological *and* orthographic overlap. That is, both the phonology and orthography of all competitor pairs were the similar. Conversely, all extant research includes some competitor pairs with distinct spellings. Consider the example pair, EAGLE – IGLESIA (church) (Blumenfeld & Marian, 2013). These words share phonology word-initially but are orthographically very distinct. Several studies suggest that learning to read changes the way the brain processes language and the orthographic representation of spoken words is activated (Perre & Ziegler, 2008; Taft et al., 2008). Using stimuli that share both phonology and orthography may change the pattern of cross-language competition for languages that have transparent as opposed to opaque orthographies.

Another explanation for the varied results is related to participant selection, and more specifically, language dominance. Importantly, extant work has tested individuals who have a clear dominant language, acquiring their second language in adolescence or adulthood (Marian & Spivey, 1999, 2003; Sarrett et al., 2022). Conversely, participants in the current study were relatively balanced in their use of each language, demonstrated similar target word recognition, and acquired both languages early in life (before the age of 4 years).

#### 4.3.2. Written words

For written words, we asked whether a different language that shares a script is activated when a letter string is presented. Although previous research has found cross-language activation in the written modality, we failed to find effects in the current study. This is likely due to methodological differences. Previous research primarily used priming paradigms to show that the recognition of a target word is influenced by the prior presentation of an orthographically similar word from another language (Bijeljac-Babic et al., 1997; Brysbaert et al., 1999; Dijkstra et al., 1998, 1999, 2000; Doctor & Klein, 1992). However, such paradigms promote cross-language activation for several reasons. First, participants read the competitor and target word in rapid succession, and thus, priming may be achieved based solely on visual matching. For example, the Dutch word VORK could speed the recognition of the English word PORK, based on the featural overlap between the words regardless of the input language. Conversely, in the current study, participants never read the competitor word at any point in the experiment providing a rigorous test of co-activation. Second, many priming studies use cognates – words with perfect semantic overlap and significant orthographic overlap (e.g., OBEDIENCE*-*OBEDIENCIA) – which create the most likely scenarios for cross-language activation, while the current study used cohort competitors that overlap in the first consonant and vowel.

### 4.4 Comparing effects across modality

Finally, we compared cross-language effects for spoken and written words to understand the extent to which language nonselective access differs by modality. There are several possible reasons why we did not find cross-language effects for written language in the current study. One explanation is related to the different developmental timelines of spoken and written language. Bilinguals may not receive instruction in reading their minoritized language (in this case, Spanish). Consequently, they may develop stronger skills in separating their languages while reading. Moreover, it is reasonable to assume that code-switching occurs less frequently in written communication compared to spoken interaction. As a result, some bilinguals may find it easier to uphold language boundaries and prevent interference between their two languages while reading, thus minimizing cross-language activation.

Another interpretation that is not mutually exclusive is due to differences in the nature of the sensory input between spoken and written word recognition. Previous work on monolinguals shows that written words demonstrate overall weaker competition during the VWP because activation does not derive from temporary uncertainty like spoken words (Apfelbaum et al., 2023; Hendrickson et al., 2022). Rather, for written words, chunks of input (letters, bigrams etc.) are directly associated with words and whenever the bottom-up match to the input is achieved, these words are activated. This hampers competition effects for written words because by the time enough input accumulates to start to activate a competitor, the target may be quite active and exert inhibitory effects on competitors (Hendrickson et al., 2022). Consistent with this finding, we replicate this pattern when comparing within-language trials for spoken and written words. This combined with the fact that cross-language effects are universally weaker than within-language effects may result in a failure to find cross-language effects for written words in the current study. From this view, the temporary ambiguity that is inherent in processing spoken words is paramount for language nonselective access, largely attenuating any effects of shared language forms.

## 5. Conclusion

Together, our results show that individuals who can understand, speak and read in two languages display evidence of automatic, incremental and parallel processing when listening and reading. Bilingual and biliterate adults build word representations bit-by-bit overtime. Nevertheless, how individuals negotiate two language systems to build lexical representations in the moment differs across modality. Bilinguals may exhibit enhanced language separation abilities in written language, possibly influenced by factors such as reduced code-switching and limited direct reading instruction in Spanish. Furthermore, for spoken words, the signal unfolds overtime giving an additional boost to within- and cross-language competition, whereas written words can be seen all at once, and thus, incremental processing is less of a factor, which results in less competition within a language and no competition across languages.

Our results are in line with the view that lexical access is fundamentally nonselective but can behave in selective ways under more constrained circumstances (Dijkstra & Kroll, 2005). Some tasks (e.g., priming paradigms with cognates), may serve to boost language nonselective access, while more constrained tasks (e.g., the current task) may make language-specific access more probable by requiring individuals to access the semantic representation of a cross-language competitor solely from the target word form. The constraint of the task may interact with the modality of the input: spoken words can elicit cross-language effects in highly constrained tasks because the signal itself draws on incremental processing, which always boosts competition, whereas written words may fail to show effects in highly constrained tasks because there is overall less lexical competition due to the nature of the static signal. Moreover, language learning history plays a role such that age of acquisition and language dominance influence the pattern of competition. Thus, it is crucial to interpret results in the context of the experimental design, given that the presence or absence of language nonselectivity is highly task, context, modality and participant dependent.

## Supporting information

10.1017/S1366728925100928.sm001 Hendrickson et al. supplementary material Hendrickson et al. supplementary material

## Data Availability

The data that support the findings of this study are available from the corresponding author upon reasonable request. Any restrictions on data availability are due to ethical or privacy considerations.

## Supplementary material

The supplementary material for this article can be found at http://doi.org/10.1017/S1366728925100928.
