# Supplementary material for: Language nonselective lexical access in bilinguals: Input modality matters
Source: Biling (Camb Engl). 2026 Jan 15:1–14. Online ahead of print. doi: 10.1017/S1366728925100928 (PMC12885054; doi:10.1017/S1366728925100928)
Supplement: Hendrickson et al. supplementary material [file S1366728925100928sup001.pdf]

Table S1. Experimental Stimuli. The experimental stimuli are shown in the tables below, with word translations provided in parentheses. The first table presents the word sets used in the English block, and the second shows those used in the Spanish block. Within each block, all words were presented exclusively in that language. Word pairs were grouped into sets of four words, such that one pair served as the unrelated items for the other pair. Each word pair appeared in only one of the four experimental conditions: English to Spanish (E–S), English to English (E–E), Spanish to English (S–E), or Spanish to Spanish (S–S).

**English Block**				
**Set**	**Word Pair**	**Competitor 1**	**Competitor 2**	**Condition**
1	1	dedo (finger)	deck (plataforma)	E-S
	2	word (palabra)	worship (adoración)	E-E
2	3	lengua (tongue)	length (longitud)	E-S
	4	mail (correo)	maid (doncella)	E-E
3	5	revista (magazine)	referee (arbitro)	E-S
	6	pouting (puchero)	power (poder)	E-E
4	7	concha (shell)	corner (esquina)	E-S
	8	table (mesa)	tailor (costurero)	E-E
5	9	pelo (hair)	pencil (lápiz)	E-S
	10	turtle (tortuga)	turkey (pavo)	E-E
6	11	noche (night)	notebook (cuaderno)	E-S
	12	bracelet (pulsera)	braid (trenza)	E-E
7	13	pesa (weight)	penny (centavo)	E-S
	14	clown (payaso)	cloud (nube)	E-E
8	15	doce (twelve)	dough (masa)	E-S
	16	rake (rastrillo)	rain (lluvia)	E-E
9	17	boda (wedding)	bones (huesos)	E-S
	18	plank (tabla)	plane (avión)	E-E
10	19	boca (mouth)	bowl (plato ondo)	E-S
	20	nail (clavo)	claw (garra)	E-S

11	21	piso (floor)	pink (rosado)	E-S
	22	carrot (zanahoria)	carriage (carroza)	E-E
12	23	cobija (blanket)	coal (carbón)	E-S
	24	funnel (embudo)	fungus (hongo)	E-E
13	25	prestar (borrow)	present (regalo)	E-S
	26	mustard (mostaza)	mustache (bigote)	E-E
14	27	pollo (chicken)	port (puerto)	E-S
	28	teacher (maestra)	team (equipo)	E-E
15	29	dormir (to sleep)	doorway (entrada)	E-S
	30	candle (vela)	canister (frasco)	E-E
16	31	bolsa (bag)	boulder (roca)	E-S
	32	tinfoil (aluminio)	tissue (pañuelo)	E-E
17	33	partido (match)	party (fiesta)	E-S
	34	bubble (burbuja)	bucket (cubeta)	E-E
18	35	jardín (garden)	garbage (basura)	E-E
	36	money (dinero)	mother (madre)	E-E
19	37	caras (faces)	carpet (alfombra)	E-S
	38	bunny (conejo)	button (botón)	E-E
20	39	leche (milk)	leg (pierna)	E-S
	40	toad (sapo)	token (moneda)	E-E

**Spanish Block**				
**Set**	**Word Pair**	**Competitor 1**	**Competitor 2**	**Condition**
1	1	risa (laugh)	ring (anillo)	S-E
	2	cuello (neck)	cuento (story)	S-S
2	3	bosque (forest)	border (frontera)	S-E
	4	cielo (sky)	ciencia (science)	S-S
3	5	coro (choir)	core (centro)	S-E

	6	paleta (ice cream)	paloma (dove)	S-S
4	7	faro (lighthouse)	farm (granja)	S-E
	8	cinta (band)	cinco (five)	S-S
5	9	lobo (wolf)	loaf (pan)	S-E
	10	viento (wind)	vientre (uterus)	S-S
6	11	ola (wave)	old (viejo)	S-E
	12	pecho (chest)	perro (dog)	S-S
7	13	barco (boat)	bark (corteza)	S-E
	14	estufa (stove)	estrella (star)	S-S
8	15	gato (cat)	garlic (ajo)	S-E
	16	cerveza (beer)	cerradura (lock)	S-S
9	17	sangre (blood)	sandy (arenoso)	S-E
	18	hombre (man)	hombro (shoulder)	S-S
10	19	calor (hot)	card (tarjeta)	S-E
	20	desayuno (breakfast)	despacho (office)	S-S
11	21	plancha (iron)	plaster (yeso)	S-E
	22	gordo (fat)	gorra (cap)	S-S
12	23	conejo (rabbit)	comb (peine)	S-E
	24	abrigo (jacket)	abrazar (to hug)	S-S
13	25	celos (jealousy)	cellar (cava)	S-E
	26	rojo (red)	ropa (clothes)	S-S
14	27	peluca (wig)	pepper (pimienta)	S-E
	28	carne (meat)	cartas (letters)	S-S
15	29	beca (scholarship)	belly (pansa)	S-E
	30	sombrero (hat)	sombrilla (umbrella)	S-S
16	31	sobre (envelope)	soak (remojar)	S-E

	32	gallina (hen)	galleta (cookie)	S-E
17	33	falda (skirt)	farmer (campesino)	S-E
	34	gritar (to scream)	grillo (cricket)	S-S
18	35	globo (balloon)	glow (brillo)	S-E
	36	camisa (t-shirt)	cama (bed)	S-S
19	37	goma (rubber)	gold (oro)	S-E
	38	calabaza (pumpkin)	calavera (skull)	S-S
20	39	cotorra (parrot)	coaster (portavasos)	S-E
	40	queso (cheese)	quemar (to burn)	S-S

Table S2. Counts of Word Class by Condition and Animacy

Condition	Word Class	Animacy		Total
		Yes	No	
English to English	Noun	10	27	37
	Verb	2	0	2
	Adjective	0	1	1
English to Spanish	Noun	6	30	36
	Verb	2	0	2
	Adjective	0	2	2
Spanish to English	Noun	8	27	36
	Verb	1	1	2
	Adjective	2	1	3
Spanish to Spanish	Noun	9	26	35
	Verb	2	0	2
	Adjective	1	2	3
